# Prevalence and Age-Associated Bacterial Chondronecrosis with Osteomyelitis Lesions in Commercial Broiler Flocks in Central Java, Indonesia

**DOI:** 10.3390/ani16121910

**Published:** 2026-06-19

**Authors:** Andi Asnayanti, Aji Praba Baskara, Muhsin Al Anas, Anh Dang Trieu Do, Douglas Rhoads, Adnan A. K. Alrubaye

**Affiliations:** 1 Center of Excellence for Poultry Science, University of Arkansas, Fayetteville, AR 72701, USA; aasnayan@uark.edu (A.A.); ad086@uark.edu (A.D.T.D.); 2 Faculty of Animal Science, Universitas Gadjah Mada, Yogyakarta 55281, Indonesia; aji.praba.b@mail.ugm.ac.id (A.P.B.); muhsin_alanas@ugm.ac.id (M.A.A.); 3 Cell and Molecular Biology Program, University of Arkansas, Fayetteville, AR 72701, USA; drhoads@uark.edu

**Keywords:** age, osteomyelitis, lameness, broilers, bones

## Abstract

In tropical countries, broiler chickens are exposed to elevated ambient temperatures and humidity, which are sometimes exacerbated by high stocking densities and poor litter quality, thereby predisposing birds to severe stress, weakening immune function, and promoting BCO lameness progression. BCO lameness causes tremendous economic losses to the poultry industry and increases the risk of foodborne disease. BCO is frequently underdiagnosed in live populations, resulting in an iceberg phenomenon in which subclinical lesions are more prevalent than clinically apparent lameness. Therefore, a total of 500 Cobb500 broiler chickens from five commercial broiler flocks in Central Java, Indonesia, were randomly selected, weighed, slaughtered, and necropsied to evaluate the prevalence of BCO lameness lesions in the proximal femoral and tibial heads across distinct market ages ranging from 33 to 43 days. The ambient housing temperature in the region can reach 28–29 °C during the day. The results showed that more than 80% of the samples had normal femora at 33 days of age with an average body weight of 1.9 kg. A significant increase in the frequency and severity of femoral and tibial lesions was recorded at 35 to 36 days of age, when the average body weight reached approximately 2.5 kg. The high frequency of worsening BCO lesions observed during the 5th week suggests an age-related pattern in BCO occurrence during the late stages of grow-out. These findings suggest that improvements in nutrition, environment, and production management strategies before 36 days of age are necessary to mitigate the impact of BCO lameness in the poultry industry.

## Simple Summary

This study aimed to examine the prevalence of bacterial chondronecrosis with osteomyelitis (BCO)-associated lesions in commercial broiler flocks in Indonesia, with age serving as a critical determinant of broiler growth rate. The results demonstrated that BCO-associated lesions in broilers exhibited a clear age-related pattern with a peak incidence during the late stages of grow-out. The BCO-associated lesions observed in this study indicate a high prevalence of BCO lameness in tropical production systems due to compounded stress, heat, and microbial load, highlighting the need for improved housing, ventilation, biosecurity, and preventive measures, such as feed supplementation.

## 1. Introduction

Consumption of poultry meat has increased dramatically and is expected to rise to 200 million tons by 2050 [1]. The demand for chicken meat in Indonesia is anticipated to continue growing. In 2025, the national production volume was 4 million tons [2], to which the province of Central Java contributed 787 thousand tons [2]. In the past seven decades, chickens have undergone intense selective breeding, resulting in fast-growing strains [3,4]. Despite their high feed efficiency, fast-growing chickens may fail to achieve proper functional maturation of organs, such as in the cardio-pulmonary system and skeletal bones, leading to metabolic disorders and locomotion abnormalities [5,6,7,8,9,10,11,12]. In particular, fast-growing broilers are vulnerable to bacterial chondronecrosis with osteomyelitis (BCO) lameness, a skeletal disease owing to rapid mass accretion that is incommensurate with skeletal growth [13,14]. Excessive body weight imposes torque and shear stress on structural bones, particularly on the proximal femora, proximal tibiae, and flexible thoracic vertebrae [14,15]. The ensuing physiological stress further weakens the epithelial barriers in the intestinal and respiratory tracts, thereby inducing leaky gut symptoms and severe bacteremia. Ultimately, pathogens in the bloodstream reach and colonize the growth plate of the long bones, causing severe osteomyelitis and bone necrosis [16,17,18]. BCO is a leading communicable disease causing lameness in broilers [14,19].

Elevated ambient temperature, high bird density, poor litter quality, and poor hygiene practices aggravate the risk of skeletal abnormalities in commercial broiler populations [19,20]. A previous study reported a higher lameness rate under high stocking densities [21]. Furthermore, chickens experiencing long-term heat stress (HS) have low levels of thyroid hormones, which decreases their metabolic rate and heat production [22,23]. HS induces immunosuppressive effects by raising the heterophil-to-lymphocyte ratio and promotes neuroendocrine responses by activating the hypothalamic–pituitary gland to release corticosterone [24,25,26]. Corticosterone acts as a stress countermeasure, compromising energy metabolism for developmental and reproductive processes and ultimately reducing feed efficiency and growth performance [26,27,28,29]. HS also induces elevated oxidative stress and inflammatory responses. Oxidative stress occurs when reactive oxygen species (ROS) exceed antioxidant defenses (e.g., glutathione peroxidase, catalase, and superoxide dismutase), causing membrane lipid peroxidation, protein and DNA denaturation, and eventually driving cell apoptosis and tissue necrosis [30]. With regard to litter quality, a previous study reported an increase in lameness cases among birds reared on reused, poor-quality litter compared to those reared on new litter [31]. Lame birds experience significant discomfort and pain, leaving them unable to walk or move to access water and feed. In practice, culling of BCO-afflicted birds is necessary to prevent outbreaks within flocks [14].

Postmortem examination of broilers at the 1st, 4th, and 5th weeks of age from 20 commercial farms in Australia recorded a 28% BCO lesion rate [13]. In Norway, a study of 50 broiler flocks revealed 19% moderate-to-severe lameness lesions [32]. In Bulgaria, the clinical lameness rate reached 10–15% of the total 650,000 chickens from 38 broiler flocks [33]. Despite these losses, most poultry farmers are not fully aware of the high prevalence of clinical and subclinical lameness in broilers [14], leading to an “iceberg” phenomenon in the poultry industry. Likewise, the lack of data on BCO occurrences in tropical countries constitutes a significant gap in the disease landscape in these regions. This study aimed to quantify the prevalence of age-associated BCO lesions in commercial broiler flocks in Central Java, Indonesia, representing a first step in the elucidation of BCO pathology and epidemiology in the region. The findings are essential to raise awareness of the risks of subclinical BCO lameness in broilers and to identify critical time points for potential intervention to sustain poultry production in the region.

## 2. Materials and Methods

### 2.1. Animal Use Statement

The animal study protocol was approved by the Animal Care and Use Ethics Commission, National Research and Innovation Agency, with Decree No. 071/KE.02/SK/05/2025.

### 2.2. Survey Design

**Figure 1:**
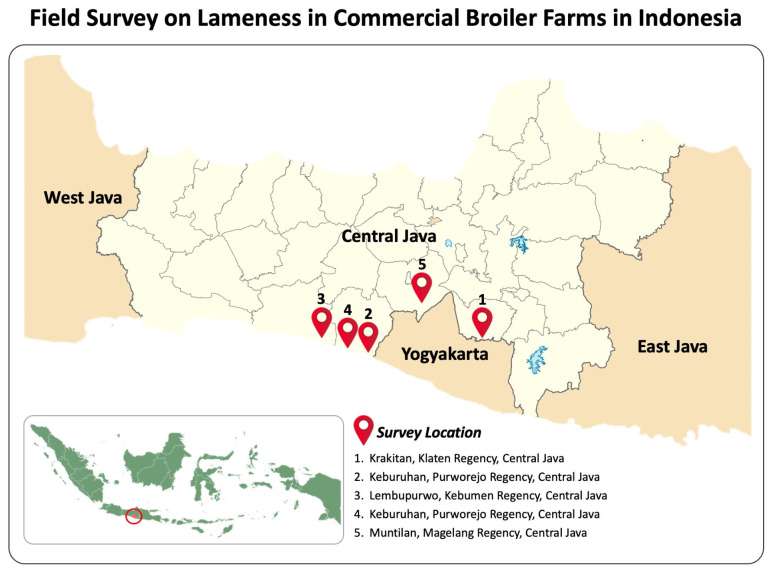
Survey sites of BCO lameness in five broiler farms in Central Java, Indonesia. The approximate geographical sampling region is circled in red. Dropped pins represent the sites of the targeted commercial farms in Krakitan, Keburuhan 1, Lembupurwo, Keburuhan 2, and Muntilan, respectively.

The survey on the prevalence of BCO-associated lesions in broilers was conducted as a cross-sectional field survey involving five independent commercial broiler flocks in Central Java in May 2025 (Figure 1). The five commercial farms in Krakitan, Keburuhan 1, Lembupurwo, Keburuhan 2, and Muntilan were selected based on their use of similar breeders, comparable commercial management practices, and the availability of flocks corresponding to the targeted market-age categories. Each flock from each farm represented a commercial market age of 33, 35, 36, 40, and 43 days, reflecting common harvesting ages in Indonesia for depopulation at 32–35 days of age and final harvest at 40–43 days of age.

**Figure 2:**
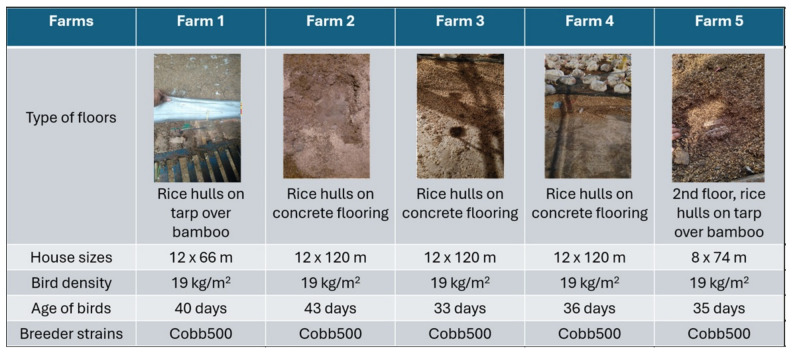
The floor types, house sizes, bird density, age, and the breeder strains used in the sampled farms.

Although the birds originated from different farms, all farms were located within the same geographical region and operated under comparable commercial management systems, including the use of Cobb500 broilers, similar stocking densities, feeding programs, and environmental conditions. The stocking density was maintained at approximately 19 kg/m^2^ by gradual depopulation (releasing the birds to the market) from 32 days of age to a final harvest age of 43 days. Birds had ad libitum access to feed and clean water. Feeding followed a standard three-phase program (starter, grower, and finisher), and the diet formulation is presented in Table 1. The breeders and diets were supplied by PT. Charoen Pokphand Indonesia Tbk, Central Java, Indonesia. The average environmental temperature in Central Java is 33–35 °C, while indoor growing houses can reach 28–29 °C during the day. There were two types of flooring used: concrete and tarp-covered bamboo flooring with rice hulls, as shown in Figure 2. Air circulation was controlled by exhaust fans in the back of the house, and lighting followed standard industry practices.

**Figure 3:**
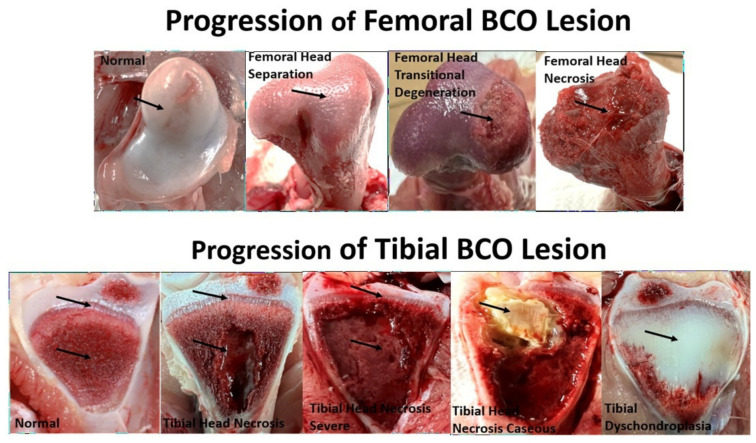
Categorization of femoral (**top**) and tibial (**bottom**) lesion progressions. Femoral head and proximal tibia appear entirely normal (N); proximal femoral head separation (FHS) or epiphyseolysis; proximal femoral head transitional (FHT) degeneration; proximal femoral head necrosis (FHN); proximal tibial head necrosis (THN); proximal tibial head necrosis severe (THNS); proximal tibial head necrosis caseous (THNC); and tibial dyschondroplasia (TD) [35]. Arrows represent the progression of the lesion from the normal femur and tibia to the most severe femoral lesion (FHN) and the most severe tibial lesion (THNC), respectively (from left to the right figures). In the case of TD, the arrow signifies abnormal cartilaginous mass in place of cancellous bone tissue.

The sample size was 0.5% for each farm, representing *n* = 100 birds per flock with a total population of 20,000 birds. The overall sample was 500 birds from five farms. Birds were randomly selected from multiple sites throughout the house without preference for body weight, clinical lameness, or visible health status to minimize sampling bias. The body weight of the birds was recorded, and the birds were subsequently slaughtered for individual necropsy to diagnose BCO lesions of the proximal tibial and femoral heads. The severity level of the lesions was scored according to the classification presented in Figure 3 [20,34].

### 2.3. Statistical Analyses

For analysis, the femoral and tibial lesions were quantitatively scored, as presented in Table 2.

The BW and frequency of bone lesions were calculated as simple frequency statistics using Microsoft Excel (Microsoft Corporation, Redmond, WA, USA) and further analyzed in JMP Student Edition 19 (SAS Institute, Cary, NC, USA). The comparison of BW between ages was analyzed using the nonparametric Kruskal–Wallis rank sum test followed by a post hoc Dunn’s test. Logistic regression was applied to lesion scores and BW. Statistical significance was determined at *p* < 0.05.

## 3. Results

### 3.1. Broiler BW, Age, and Lesion Scores

**Figure 4:**
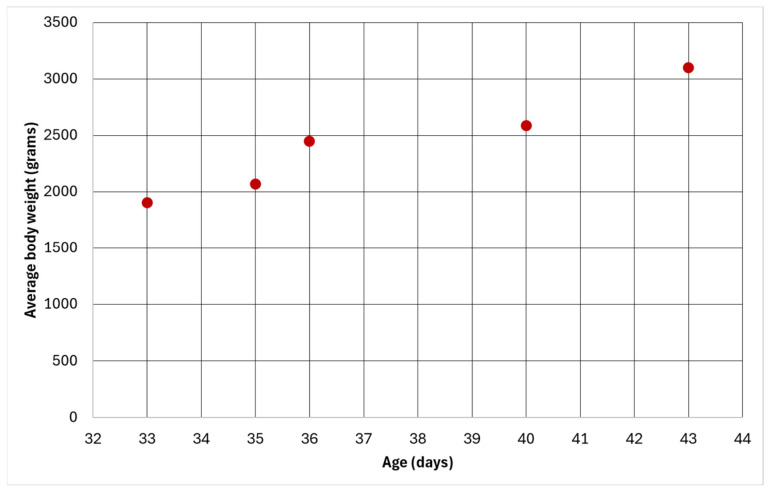
The distribution of BW across populations by age.

The distribution of BW data across the age groups of the sampled Cobb500 broilers is visualized in Figure 4.

In general, broiler BW increased with age. The average BW and lesion score comparisons across population age groups are detailed in Table 3. Femoral and tibial lesions significantly increased from 35 to 36 days of age, when the birds reached an average BW of approximately 2.5 kg.

### 3.2. Lameness Lesions

**Figure 5:**
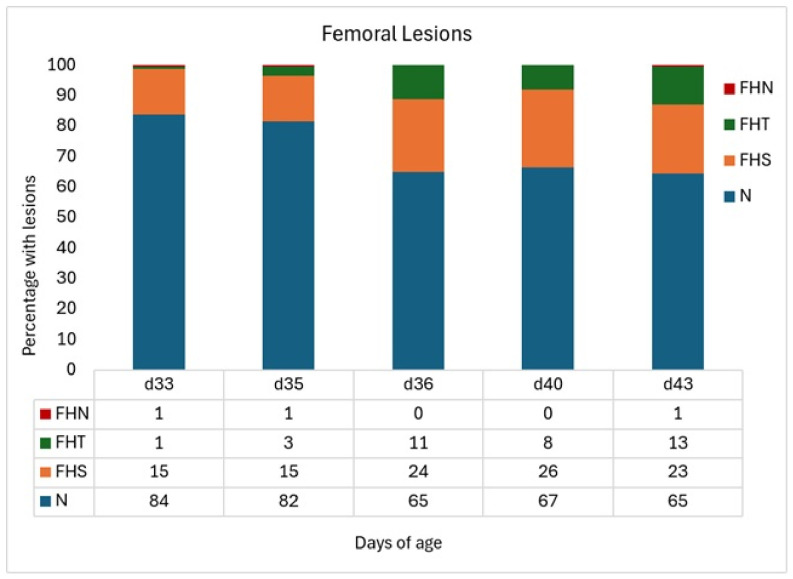
Percentage of femoral lesions in each category. N = normal femoral head; FHS = proximal femoral head separation; FHT = proximal femoral head transitional degeneration; and FHN = proximal femoral head necrosis. Of the five flocks, N and FHS were the most and second-most prevalent lesion categories, respectively.

The severity of lameness lesions in the legs was examined by necropsy of the femoral head and proximal tibia. A comparison of femoral lesions across different ages is presented in Figure 5. At 33 days of age, the cumulative prevalence of normal femoral heads (N) was greater than 80%; thereafter, the percentage of N decreased with age. Noticeably, femoral lesion prevalence—particularly FHS and FHT—increased from 35 to 36 days of age, then remained relatively constant until 43 days of age (Figure 5). At 43 days of age, the average prevalence of N, FHS, FHT, and FHN was 65%, 22.5%, 12.5%, and 0.5%.

**Figure 6:**
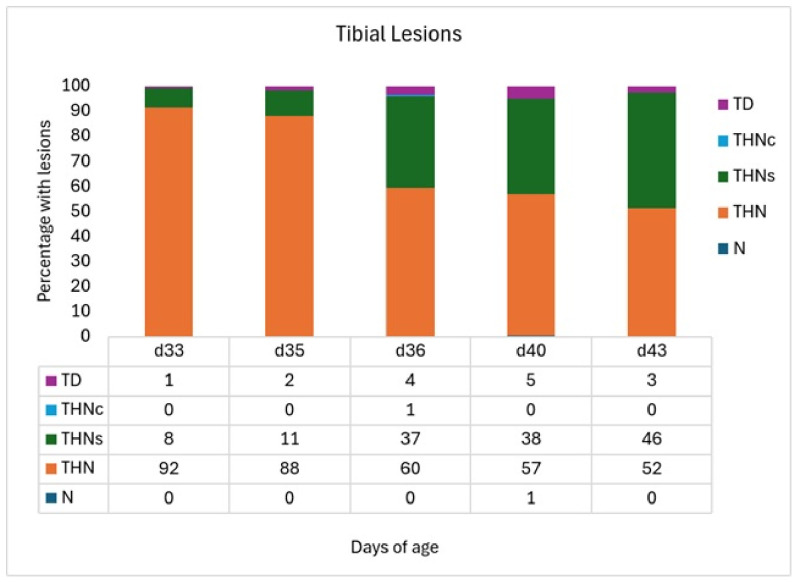
Percentage of tibial lesion categories. N = normal tibial head; THN = proximal tibial head necrosis; THN_C_ = proximal tibial head necrosis caseous; THN_S_ = proximal tibial head necrosis severe; TD = tibial dyschondroplasia. Of the five flocks, THN and THN_S_ lesions were the most and second-most prevalent categories, respectively.

A comparison of tibial lesions across distinct ages is presented in Figure 6. There were no normal proximal tibiae (N) diagnosed at 33 days of age. THN lesions (92%) were the most prevalent, followed by THNS lesions (8%). A significant increase in THNS lesions was observed from 35 to 36 days of age and ultimately reached 46% by 43 days of age. Several TD cases (3%) were also recorded starting at 36 days of age.

## 4. Discussion

This study aimed to assess the average BW and the prevalence of BCO-associated lesions in commercial broiler flocks across different ages in Central Java, Indonesia. The average BW of Cobb500 broilers at 33, 35, 36, 40, and 43 days of age was approximately 1.9 kg, 2.1 kg, 2.5 kg, 2.6 kg, and 3.1 kg, respectively. Overall, these BW records are lower than the standard BW of Cobb500 at the same time points reported by Cobb in the Broiler Performance and Nutrition Supplement in 2022 [35]. Generally, the genetics of the breeders, nutrition, and grow-out management, including temperature and flooring, are critical factors determining the performance and BW of broilers [36,37]. Several factors pertinent to the region of study may have contributed to this lower broiler weight. The administered feed followed the standard commercial formula; however, its ingredient sources differed. In addition, rearing temperatures reaching up to 28–29 °C potentially led to heat stress and subpar BW accretion due to a reduced growth rate.

Sampling of broilers at 33, 35, 36, 40, and 43 days of age was conducted based on both commercial and biological considerations. From an industrial perspective, these ages represent common harvesting points for broiler chickens in Indonesia, where depopulation is performed to maintain an optimal stocking density of 19 kg/m^2^ at 32–35 days of age, and the remaining birds are marketed at 40–43 days of age. From a biological standpoint, these ages encompass the period of rapid weight gain and increasing skeletal load, which has been consistently associated with elevated susceptibility to BCO [38]. Therefore, the selected age points allowed us to evaluate lesion prevalence before, during, and after the anticipated critical period of BCO progression. It should be noted that although the birds were sampled from different farms, they were from the same geographical region and environmental conditions, with similar production management. Thus, differences in flooring types (concrete and tarp-covered bamboo) were negligible in the evaluation of lesions across farms, as such flooring systems are not known to exhibit any undue physiological stress on broiler legs [20]. Furthermore, since management practices among participating farms were generally similar and the study was not specifically designed to evaluate environmental risk factors, these variables were not incorporated into the statistical model.

At 33 days of age, more than 80% of the femora were categorized as normal, with no visible damage. It is important to note that most of the sampled broilers appeared clinically healthy at this age. Theoretically, chickens reach the peak of their immunity levels at 30 to 34 days of age [39]. However, approximately 90% of the tibiae showed mild tibial lesions (THN). The presence of THN lesions in clinically healthy birds is not necessarily associated with BCO lameness but rather attributed to osteochondrotic clefts during fast growth plate proliferation [14,40,41]. Naturally, mechanical pressure on the growth plate induces the formation of osteochondrotic clefts in the cartilage layers, which are essential for the survival and maturation of chondrocytes [14,40,41]. Thus, osteochondrotic microfractures provide ideal niches for the settlement of hematogenously circulated pathogens.

Severity levels of the lesions significantly increased from 35 to 36 days of age (femora: FHS to FHT and tibiae: THN to THNS), and the proportion of the lesions remained approximately the same until 43 days of age. Several studies have reported the prevalence of BCO in commercial broiler flocks aged 4 to 8 weeks, with the peak occurrence rate happening at 5 weeks of age [14,42,43,44,45,46]. The peak incidence of BCO corresponds to significant body weight accumulation [18]. The frequency of osteochondrotic lesions is presumed to increase when body mass accumulation—approximately 2.5 kg in this study—is adequate to impose excessive mechanical force on the structural bones. Pressures from mechanical stress and BW required for femoral damage were inadequate to induce severe FHN lesions in this study [47]. If the birds experience additional stressors, such as extreme heat, BCO progression can occur rapidly, leading to severe necrosis (FHN) and ultimate mortality within 24–48 h [46].

In addition to high BW stress, another possible predisposing factor to the increasing severity of BCO-associated lesions is elevated house temperature. A previous study demonstrated substantially higher subclinical incidences of tibial head necrosis (THN) in heat-stressed broilers compared with broilers reared under thermoneutral conditions [15]. HS deteriorates skeletal bone health through multiple pathways, particularly by (1) impairing functional maturation of T and B cells in lymphoid tissues and inducing inflammatory cytokines [48]; (2) disrupting calcium and mineral resorption in the gastrointestinal tract [49]; (3) inducing high bacteremia and pathogen translocation to the skeletal bones [50]; (4) inducing cell apoptosis and bone necrosis due to high oxidative stress responses [30]; and (5) reducing the formation of bone cells and promoting bone resorption as a consequence of excessive glucocorticoids [24,25,26,27,28,29,51].

While osteochondrotic clefts provide ideal niches for bacterial colonization in the bone, stress-mediated immunosuppression promotes hematogenous distribution of pathogens to reach and sustain infection in the aforementioned niches. Bacterial invasion may secrete fibrinonecrotic exudate in the metaphyseal zone, expanding the lytic activity to microfractures in the growth plate and aggravating the THN lesion to THNS [20]. In the femur, bacterial necrosis underlying the fractured remnant of the femoral head induces progressive necrosis, ulceration, erosion, and fracturing of the growth plate, resulting in femoral head transitional degeneration (FHT) [20]. At the 5th week (36 days of age), the average of the severe tibial (THNS) and femoral lesions (FHS, FHT, and FHN) reached 38.5%, which is higher than the BCO rate reported in Australia (28%) [13]. Overall, the elevated temperature signifies the high rate of BCO lesions recorded in this study. In addition, several TD cases were observed at 36 to 43 days of age, indicating symptoms of imbalanced nutrition, particularly low levels of calcium and high levels of phosphorus [52,53].

Several management practices in broiler farms can hinder the activity of birds, such as low light intensities, high stocking densities, and easy access to drinkers and feeders [54,55]. Less activity significantly obstructs the blood supply to epiphyseal and physeal cartilage, which ultimately triggers osteochondrosis in the bones [56]. In this survey, bird density is unlikely to be a significant factor in the progression of BCO lameness lesions since the production management strictly maintained stocking density at approximately 19 kg/m^2^.

Overall, this study underscores the prevalence of moderate BCO lesions in clinically healthy birds, which represent subclinical BCO lesions that can progress to severe clinical BCO lameness when an extreme stressor is present. This finding is particularly relevant to tropical production systems, where extreme temperatures may exacerbate immune suppression and skeletal stress in fast-growing broilers. Furthermore, the observed lesion progression pattern in this study is consistent with the well-established biological relationship between age, body weight accumulation, skeletal load, and BCO development reported in previous studies [46,57]. Therefore, the findings support the interpretation that age-associated physiological changes contributed substantially to the observed increase in lesion prevalence. The notable increase in lesion severity observed between 35 and 36 days of age further supports the biological relevance of these sampling points and suggests a critical intervention window before 36 days of age. Mitigation measures, particularly dietary supplements such as probiotics, prebiotics, microalgae, organic trace minerals (zinc, manganese, and copper), phytogenic, and vitamin D3 metabolic forms [19,47,58,59,60,61,62,63], and vaccination [64,65] have been intensively investigated to control BCO-induced lameness. Dietary supplementation is considered a strategic measure to control BCO lameness in broilers because of its potential for large-scale application without extra animal handling that can impose stressful conditions on the birds [38]. Following up on this publication, a comprehensive study on the bacterial etiological agents isolated from BCO lame birds in this region will be published in a separate article. Furthermore, future studies are suggested to incorporate detailed environmental monitoring to quantify the contribution of environmental stressors to BCO progression in tropical production systems.

## 5. Conclusion

The prevalence of BCO-associated lesions in commercial broiler flocks reared in tropical regions demonstrated a significant increase in BCO lesion frequency and severity levels from 35 to 36 days of age, when the BW of birds reached approximately 2.5 kg. The lameness survey conducted here is, to our knowledge, the first in the field in this geographical region. As such, these data present a critical intervention window before the age of 36 days. Mitigation measures, particularly dietary supplementation of probiotics and phytogenics, can be efficacious and practical intervention measures to mitigate BCO lameness progression in broilers.

## Figures and Tables

**Table 1:** Nutrient composition and ingredients of diets used by broiler farmers.

Nutrient Content	S00 Pre-Starter 1–7 Days	S11 Starter 8–21 Days	S12G Finisher > 21 Days
Moisture (max) (%)	14.0	14.0	14.0
Crude protein (min) (%)	22.0	20.0	19.0
Crude fat (min) (%)	5.0	5.0	5.0
Crude fiber (max) (%)	4.0	5.0	6.0
Ash (max) (%)	8.0	8.0	8.0
Ca (%)	0.8–1.1	0.8–1.1	0.8–1.1
P available with phytase enzyme (%)	0.6	0.6	0.6
Lysine (min) (%)	1.3	1.2	1.05
Methionine (min) (%)	0.5	0.45	0.4
Methionine + cysteine (min) (%)	0.9	0.8	0.75
Tryptophan (min) (%)	0.2	0.19	0.18
Threonine (min) (%)	0.8	0.75	0.65
Feedstuff	Maize, rice bran, soybean meal, wheat bran, meat and bone meal, full-fat soybean meal, and palm kernel meal	Maize, rice bran, soybean meal, wheat bran, meat and bone meal, full-fat soybean meal, and palm kernel meal	Maize, rice bran, soybean meal, wheat bran, meat and bone meal, full-fat soybean meal, and palm kernel meal
Feed additive	Vitamin, enzyme, Ca, P, and mineral	Vitamin, enzyme, Ca, P, and mineral	Vitamin, enzyme, Ca, P, mineral

Note: All feed was produced by PT. Charoen Pokphand Indonesia Tbk, Central Java, Indonesia.

**Table 2:** Quantitative scores of the femoral and tibial lesions.

Scores	0	1	2	3	4
Femoral lesions	N	FHT	FHS	FHN	-
Tibial lesion	N	THN	THNS	THNC	TD

**Table 3:** Average BW (in kg) and bone-type lesion scores, including both legs, and average per bird.

Bird Age	Average BW (kg) *	Left Tibia Score	Left Femur Score	Right Tibia Score	Right Femur Score	Average Tibia Score	Average Femur Score
33	1904	1.09 ± 0.386 ^a^	0.47 ± 0.864 ^a^	1.10 ± 0.302 ^a^	0.18 ± 0.572 ^a^	1.09 ± 0.299 ^a^	0.32 ± 0.582 ^a^
35	2068	1.13 ± 0.485 ^a^	0.36 ± 0.772 ^a^	1.17 ± 0.451 ^a^	0.33 ± 0.726 ^ab^	1.15 ± 0.417 ^a^	0.35 ± 0.626 ^a^
36	2448	1.49 ± 0.759 ^b^	0.58 ± 0.855 ^ab^	1.47 ± 0.611 ^b^	0.60 ± 0.853 ^bc^	1.48 ± 0.598 ^b^	0.59 ± 0.637 ^b^
40	2588	1.47 ± 0.758 ^b^	0.5 ± 0.810 ^ab^	1.58 ± 0.741 ^b^	0.68 ± 0.920 ^c^	1.53 ± 0.712 ^b^	0.59 ± 0.750 ^ab^
43	3098	1.51 ± 0.659 ^b^	0.81 ± 0.929 ^b^	1.56 ± 0.608 ^b^	0.37 ± 0.706 ^abc^	1.54 ± 0.578 ^b^	0.59 ± 0.708 ^b^

* Average BW provided per production house at each location, with no SD calculated/recorded. Non-connecting superscript letters denote significant statistical differences (*p* < 0.05).

## Data Availability

The dataset used and/or analyzed in the study is available from the corresponding author upon request (Adnan Alrubaye).

## References

[1] Alexandratos N., Bruinsma J. (2012). World Agriculture Towards 2030/2050: The 2012 Revision.

[2] Badan Pusat Statistik (2026). Jumlah Produksi Daging Unggas Menurut Provinsi dan Jenis (kg), 2025.

[3] Schmidt C.J., Persia M., Feierstein E., Kingham B., Saylor W. (2009). Comparison of a modern broiler line and a heritage line unselected since the 1950s. Poult. Sci..

[4] Siegel P., Dunnington E. (1997). Genetic selection strategies–population genetics. Poult. Sci..

[5] Lilburn M.S. (1994). Skeletal growth of commercial poultry species. Poult. Sci..

[6] Rath N., Huff G.R., Huff W.E., Balog J.M. (2000). Factors regulating bone maturity and strength in poultry. Poult. Sci..

[7] Scheele C. (1997). Pathological changes in metabolism of poultry related to increasing production levels. Vet. Q..

[8] Olkowski A. (2007). Pathophysiology of heart failure in broiler chickens: Structural, biochemical, and molecular characteristics. Poult. Sci..

[9] Cheema M.A. (2003). A Comparison of the Immune Performance of Commercial Growth Selected Broiler Genotypes. Master’s Thesis.

[10] Emmerson D. (2003). Breeding objectives and selection strategies for broiler production. Poultry Breeding, Genetics and Biotechnology.

[11] Kalmar I.D., Vanrompay D., Janssens G.P. (2013). Broiler ascites syndrome: Collateral damage from efficient feed to meat conversion. Vet. J..

[12] Hartcher K., Lum H. (2020). Genetic selection of broilers and welfare consequences: A review. World’s Poult. Sci. J..

[13] Wijesurendra D.S., Chamings A.N., Bushell R.N., Rourke D.O., Stevenson M., Marenda M.S., Noormohammadi A.H., Stent A. (2017). Pathological and microbiological investigations into cases of bacterial chondronecrosis and osteomyelitis in broiler poultry. Avian Pathol..

[14] Wideman R.F. (2016). Bacterial chondronecrosis with osteomyelitis and lameness in broilers: A review. Poult. Sci..

[15] Wideman R.F., Pevzner I. (2012). Dexamethasone triggers lameness associated with necrosis of the proximal tibial head and proximal femoral head in broilers. Poult. Sci..

[16] Al-Rubaye A.A., Ekesi N.S., Zaki S., Emami N.K., Wideman R.F., Rhoads D.D. (2017). Chondronecrosis with osteomyelitis in broilers: Further defining a bacterial challenge model using the wire flooring model. Poult. Sci..

[17] Butterworth A. (1999). Infectious components of broiler lameness: A review. World’s Poult. Sci. J..

[18] McNamee P.T., Smyth J.A. (2000). Bacterial chondronecrosis with osteomyelitis (‘femoral head necrosis’) of broiler chickens: A review. Avian Pathol..

[19] Alrubaye A.A., Ekesi N.S., Hasan A., Elkins E., Ojha S., Zaki S., Dridi S., Wideman R.F., Rebollo M.A., Rhoads D.D. (2020). Chondronecrosis with osteomyelitis in broilers: Further defining lameness-inducing models with wire or litter flooring to evaluate protection with organic trace minerals. Poult. Sci..

[20] Wideman R.F., Prisby R.D. (2013). Bone circulatory disturbances in the development of spontaneous bacterial chondronecrosis with osteomyelitis: A translational model for the pathogenesis of femoral head necrosis. Front. Endocrinol..

[21] Sanotra G.S., Lawson L.G., Vestergaard K.S., Thomsen M.G. (2001). Influence of stocking density on tonic immobility, lameness, and tibial dyschondroplasia in broilers. J. Appl. Anim. Welf. Sci..

[22] Pritchett E.M., Van Goor A., Schneider B.K., Young M., Lamont S.J., Schmidt C.J. (2023). Chicken pituitary transcriptomic responses to acute heat stress. Mol. Biol. Rep..

[23] Osti R., Bhattarai D., Zhou D. (2017). Climatic variation: Effects on stress levels, feed intake, and bodyweight of broilers. Braz. J. Poult. Sci..

[24] Marissal-Arvy N., Gaumont A., Langlois A., Dabertrand F., Bouchecareilh M., Tridon C., Mormede P. (2007). Strain differences in hypothalamic–pituitary–adrenocortical axis function and adipogenic effects of corticosterone in rats. J. Endocrinol..

[25] Yang J., Liu L., Sheikhahmadi A., Wang Y., Li C., Jiao H., Lin H., Song Z. (2015). Effects of corticosterone and dietary energy on immune function of broiler chickens. PLoS ONE.

[26] Liu C., Fu J., Xu F., Wang X., Li S. (2015). The role of heat shock proteins in oxidative stress damage induced by Se deficiency in chicken livers. Biometals.

[27] Oni A.I., Adeleye O.O., Adebowale T.O., Oke O.E. (2024). The role of phytogenic feed additives in stress mitigation in broiler chickens. J. Anim. Physiol. Anim. Nutr..

[28] Olfati A., Mojtahedin A., Sadeghi T., Akbari M., Martínez-Pastor F. (2018). Comparison of growth performance and immune responses of broiler chicks reared under heat stress, cold stress and thermoneutral conditions. Span. J. Agric. Res..

[29] Ahmadi F., Suleria H.A., Dunshea F.R. (2025). Role of plant bioactive compounds in improving ruminant resilience to heat stress challenge. Anim. Prod. Sci..

[30] Nan J., Yang H., Rong L., Jia Z., Yang S., Li S. (2023). Transcriptome analysis of multiple tissues reveals the potential mechanism of death under acute heat stress in chicken. BMC Genom..

[31] Paz I.C.L.A., Garcia R.G., Bernardi R., Seno L.d.O., Naeaes I.d.A., Caldara F.R. (2013). Locomotor problems in broilers reared on new and re-used litter. Ital. J. Anim. Sci..

[32] Granquist E.G., Vasdal G., De Jong I.C., Moe R.O. (2019). Lameness and its relationship with health and production measures in broiler chickens. Animal.

[33] Dinev I. (2009). Clinical and morphological investigations on the prevalence of lameness associated with femoral head necrosis in broilers. Br. Poult. Sci..

[34] Wideman J.R.F., Hamal K.R., Stark J.M., Blankenship J., Lester H., Mitchell K.N., Lorenzoni G., Pevzner I. (2012). A wire-flooring model for inducing lameness in broilers: Evaluation of probiotics as a prophylactic treatment. Poult. Sci..

[35] Cobb (2022). Cobb500 Broiler Performance & Nutrition Supplement (2022).

[36] Feddes J., Emmanuel E., Zuidhoft M. (2002). Broiler performance, body weight variance, feed and water intake, and carcass quality at different stocking densities. Poult. Sci..

[37] Brake J., Havenstein G., Scheideler S., Ferket P., Rives D. (1993). Relationship of sex, age, and body weight to broiler carcass yield and offal production. Poult. Sci..

[38] Asnayanti A., Do A., Alrubaye A. (2024). Microbiology, induction, and management practices to mitigate lameness caused by bacterial chondronecrosis with osteomyelitis in broiler chickens. Ger. J. Vet. Res..

[39] Song B., Tang D., Yan S., Fan H., Li G., Shahid M.S., Mahmood T., Guo Y. (2021). Effects of age on immune function in broiler chickens. J. Anim. Sci. Biotechnol..

[40] Wise D., Jennings A. (1973). The development and morphology of the growth plates of two long bones of the turkey. Res. Vet. Sci..

[41] Barrow M.V., Simpson C.F., Miller E.J. (1974). Lathyrism: A review. Q. Rev. Biol..

[42] Nairn M., Watson A. (1972). Leg weakness of poultry—A clinical and pathological characterisation. Aust. Vet. J..

[43] Thorp B., Whitehead C., Dick L., Bradbury J., Jones R., Wood A. (1993). Proximal femoral degeneration in growing broiler fowl. Avian Pathol..

[44] Thorp B.H. (1994). Skeletal disorders in the fowl: A review. Avian Pathol..

[45] Thorp B., Waddington D. (1997). Relationships between the bone pathologies, ash and mineral content of long bones in 35-day-old broiler chickens. Res. Vet. Sci..

[46] Asnayanti A., Do A.D., Alharbi K., Alrubaye A. (2024). Inducing experimental bacterial chondronecrosis with osteomyelitis lameness in broiler chickens using aerosol transmission model. Poult. Sci..

[47] Asnayanti A., Hasan A., Alharbi K., Hassan I., Bottje W., Rochell S.J., Rebollo M.A., Kidd M.T., Alrubaye A.A. (2024). Assessing the impact of Spirulina platensis and organic trace minerals on the incidence of bacterial chondronecrosis with osteomyelitis lameness in broilers using an aerosol transmission model. J. Appl. Poult. Res..

[48] Hirakawa R., Nurjanah S., Furukawa K., Murai A., Kikusato M., Nochi T., Toyomizu M. (2020). Heat stress causes immune abnormalities via massive damage to effect proliferation and differentiation of lymphocytes in broiler chickens. Front. Vet. Sci..

[49] Ait-Boulahsen A., Garlich J.D., Edens F.W. (1993). Calcium deficiency and food deprivation improve the response of chickens to acute heat stress. J. Nutr..

[50] Varasteh S., Braber S., Akbari P., Garssen J., Fink-Gremmels J. (2015). Differences in susceptibility to heat stress along the chicken intestine and the protective effects of galacto-oligosaccharides. PLoS ONE.

[51] Ahmad R., Yu Y.-H., Hsiao F.S.-H., Su C.-H., Liu H.-C., Tobin I., Zhang G., Cheng Y.-H. (2022). Influence of heat stress on poultry growth performance, intestinal inflammation, and immune function and potential mitigation by probiotics. Animals.

[52] Edwards H., Carlos A., Kasim A., Toledo R. (1999). Effects of steam pelleting and extrusion of feed on phytate phosphorus utilization in broiler chickens. Poult. Sci..

[53] Edwards H.M., Veltmann J.R. (1983). The role of calcium and phosphorus in the etiology of tibial dyschondroplasia in young chicks. J. Nutr..

[54] Foutz T., Ratterman A., Halper J. (2007). Effects of immobilization on the biomechanical properties of the broiler tibia and gastrocnemius tendon. Poult. Sci..

[55] Ruiz-Feria C., Arroyo-Villegas J., Pro-Martinez A., Bautista-Ortega J., Cortes-Cuevas A., Narciso-Gaytan C., Hernandez-Cazares A., Gallegos-Sanchez J. (2014). Effects of distance and barriers between resources on bone and tendon strength and productive performance of broiler chickens. Poult. Sci..

[56] Ytrehus B., Carlson C.S., Ekman S. (2007). Etiology and pathogenesis of osteochondrosis. Vet. Pathol..

[57] Do A.D.T., Assetova G., Asnayanti A., Akhmetzhanova A., Zhexenayeva A., Muratbayev D., Senkebayeva D., Bolkenov B., Alrubaye A. (2026). Survey of Bacterial Chondronecrosis with Osteomyelitis Lesion Incidence in Broiler Farms in Kazakhstan Regions. Animals.

[58] Alrubaye A.A., Ekesi N.S., Hasan A., Koltes D.A., Wideman R.F., Rhoads D.D. (2020). Chondronecrosis with osteomyelitis in broilers: Further defining a bacterial challenge model using standard litter flooring and protection with probiotics. Poult. Sci..

[59] Wideman R., Al-Rubaye A., Kwon Y., Blankenship J., Lester H., Mitchell K., Pevzner I., Lohrmann T., Schleifer J. (2015). Prophylactic administration of a combined prebiotic and probiotic, or therapeutic administration of enrofloxacin, to reduce the incidence of bacterial chondronecrosis with osteomyelitis in broilers. Poult. Sci..

[60] Asnayanti A., Alharbi K., Do A.D., Al-Mitib L., Bühler K., Van der Klis J.D., Gonzalez J., Kidd M.T., Alrubaye A.A. (2024). Early 1, 25-dihydroxyvitamin D3-glycosides supplementation: An efficient feeding strategy against bacterial chondronecrosis with osteomyelitis lameness in broilers assessed by using an aerosol transmission model. J. Appl. Poult. Res..

[61] Alharbi K., Asnayanti A., Do A.D.T., Perera R., Al-Mitib L., Shwani A., Rebollo M.A., Kidd M.T., Alrubaye A.A.K. (2024). Identifying Dietary Timing of Organic Trace Minerals to Reduce the Incidence of Osteomyelitis Lameness in Broiler Chickens Using the Aerosol Transmission Model. Animals.

[62] Alharbi K., Ekesi N., Hasan A., Asnayanti A., Liu J., Murugesan R., Ramirez S., Rochell S., Kidd M.T., Alrubaye A. (2024). Deoxynivalenol and fumonisin predispose broilers to bacterial chondronecrosis with osteomyelitis lameness. Poult. Sci..

[63] Do A.D.T., Anthney A., Alharbi K., Asnayanti A., Meuter A., Alrubaye A.A.K. (2024). Assessing the Impact of Spraying an Enterococcus faecium-Based Probiotic on Day-Old Broiler Chicks at Hatch on the Incidence of Bacterial Chondronecrosis with Osteomyelitis Lameness Using a Staphylococcus Challenge Model. Animals.

[64] Assumpcao A.L., Arsi K., Asnayanti A., Alharbi K.S., Do A.D., Read Q.D., Perera R., Shwani A., Hasan A., Pillai S.D. (2024). Electron-Beam-Killed Staphylococcus Vaccine Reduced Lameness in Broiler Chickens. Vaccines.

[65] Perera R., Asnayanti A., Alharbi K.S., Do A., Ben Larbi M., Anthney A.P., FV Assumpcao A.L., Arsi K., Kumar-Phillips G., Santamaria J.M. (2025). Leveraging Electron Beam-Inactivated Multi-Strain Staphylococcus Vaccine for Preventing BCO Lameness in Broiler Chickens. Vaccines.

